# P-603. Humoral Immunity Against Highly Pathogenic Avian Influenza is Low in Immunocompromised Persons and Not Augmented by Seasonal Vaccination

**DOI:** 10.1093/ofid/ofaf695.816

**Published:** 2026-01-11

**Authors:** Moreno Rodrigues, Isabella Sengsouk, Prasanthy Balasubramanian, Maggie Chahoud, Woudase Gallo, Xori Green, Aaron Tobian, William Werbel, Andrew H Karaba

**Affiliations:** Johns Hopkins, Baltimore, Maryland; Johns Hopkins University, Baltimore, Maryland; Johns Hopkins, Baltimore, Maryland; Johns Hopkins University, Baltimore, Maryland; Johns Hopkins, Baltimore, Maryland; Johns Hopkins University, Baltimore, Maryland; Johns Hopkins University, Baltimore, Maryland; Johns Hopkins University, Baltimore, Maryland; Johns Hopkins University, Baltimore, Maryland

## Abstract

**Background:**

Influenza, particularly novel zoonotic strains, causes severe disease in immunocompromised persons (ISPs). Global outbreaks of highly pathogenic avian influenza (HPAI, e.g., H5N1, H7N9) may pose similar risk to ISPs, yet anti-HPAI immune landscape and impact of vaccination are unknown.

**Figure:**
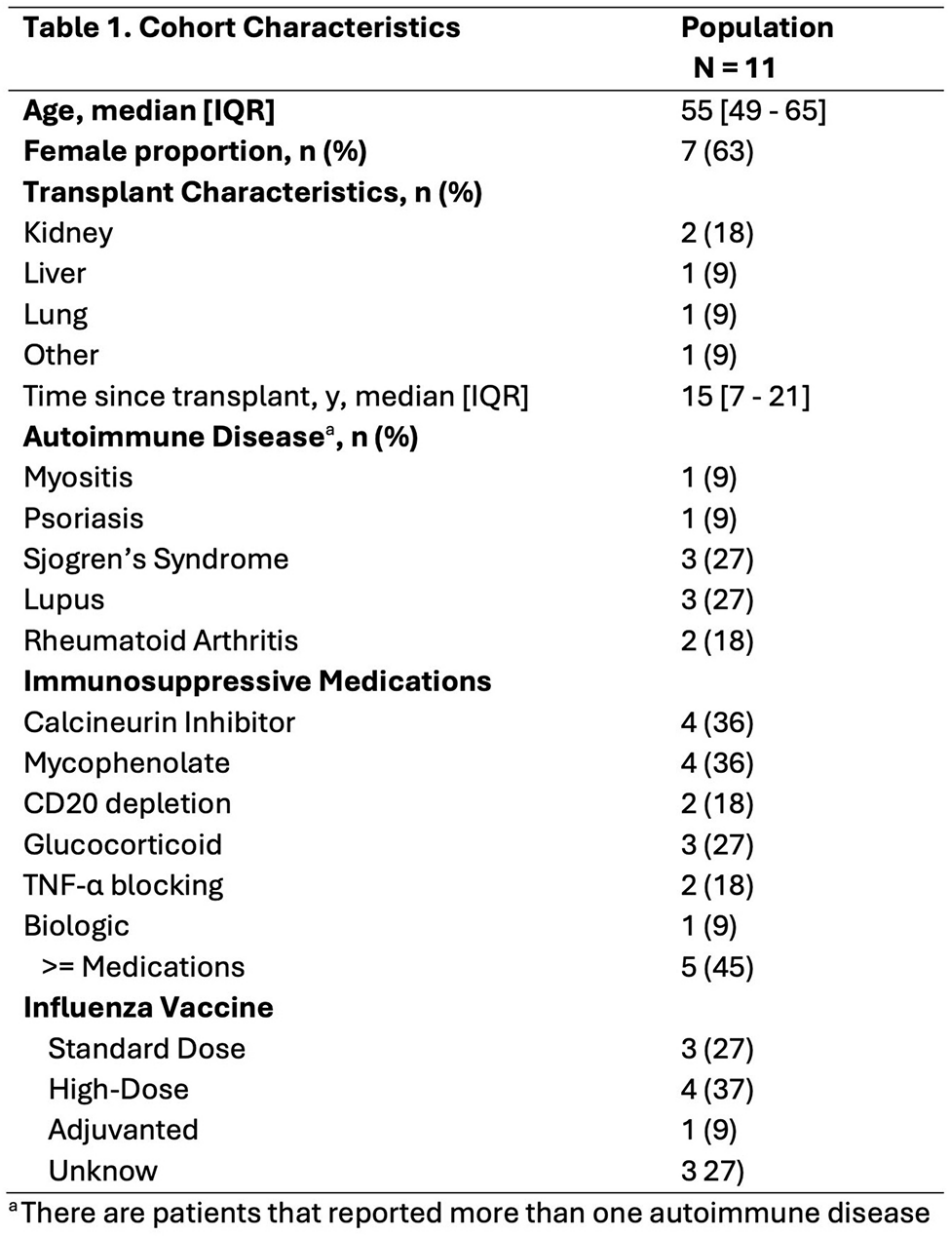

**Figure 1.**
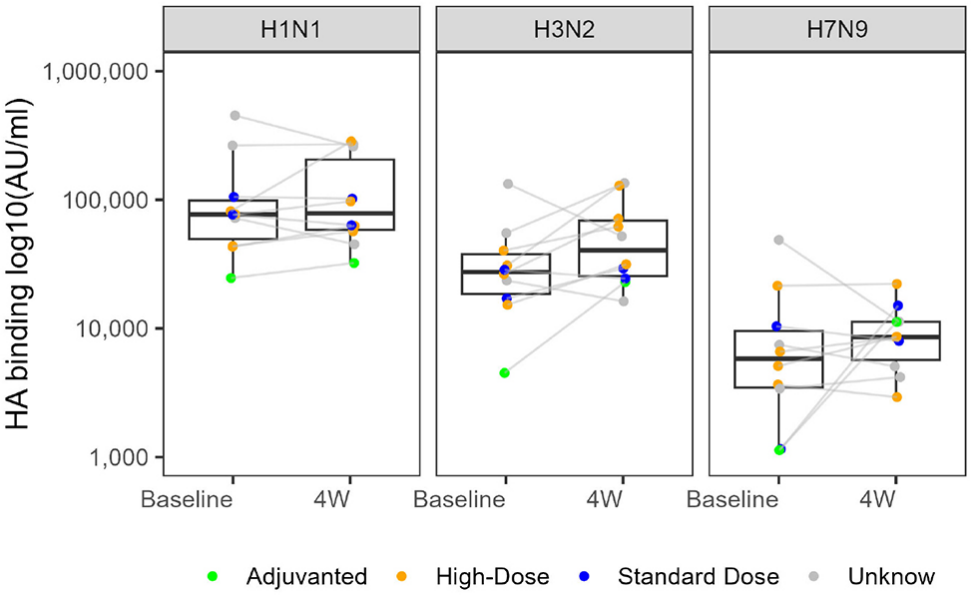
HA Binding Titers Before and After Seasonal Influenza Vaccination. Antibodies to H1, H3, and H7 were measured using the Meso Scale Discovery Respiratory Panel 4 before (baseline) and 4 weeks (4W) after seasonal influenza vaccination in immunosuppressed persons. Results are reported on the y-axis in arbitrary units (AU)/mL. Vaccine type is indicated by the color of the dot.

**Methods:**

In an ongoing national prospective cohort of ISPs reporting respiratory virus vaccination and infection, plasma from participants was analyzed for binding antibody (Ab) against seasonal (H1, H3) and HPAI (H7) hemagglutinin (HA) using a multiplex electrochemiluminescence assay (Meso Scale Discovery) and microneutralization using live contemporary vaccine strains of H1N1 and H3N2 before and after seasonal influenza vaccination. Changes in HA binding titer and neutralization were evaluated using Wilcoxon Signed Rank testing. Interstrain HA binding Pearson correlations were computed along with intrastrain HA binding and neutralization.

**Figure 2.**
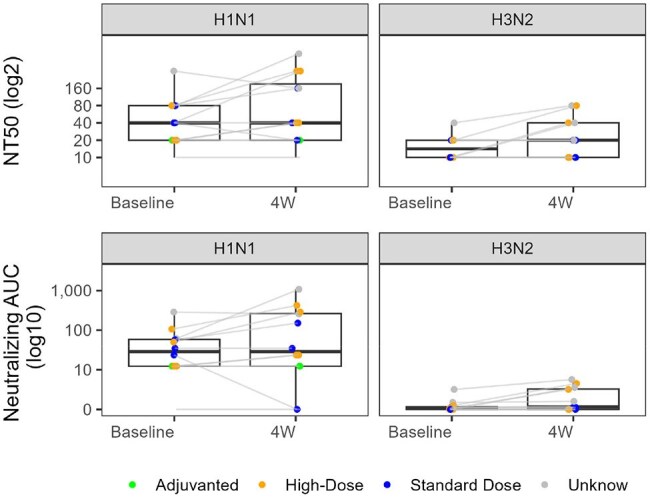
Neutralizing Titers Before and After Seasonal Influenza Vaccination. Live-virus neutralization was measured using microneutralization assay against indicated seasonal influenza strains. Top. Reciprocal of the highest plasma dilution resulting in 50% protection (NT50) at baseline and 4 weeks post vaccination. Bottom. Area under the neutralization curve (AUC) log10 transformed at baseline and 4 weeks post vaccination for vaccine strains of H1N1 and H3N2.

**Figure 3.**
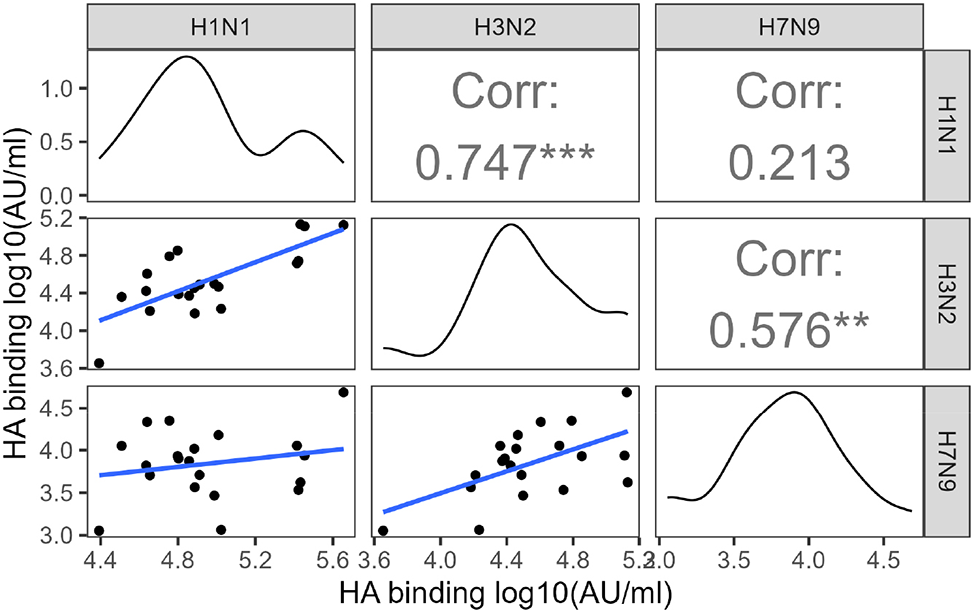
Interstrain HA Antibody Correlations. HA antibodies are plotted for each strain against one another and Pearson correlation coefficients are reported. ** and *** indicate p < 0.01 and 0.001, respectively, while no marking above the correlation coefficient indicates p > 0.05.

**Results:**

Among 11 ISPs, median [IQR] anti H1, H3, H7 titers did not change significantly from baseline (4.9 [4.7 – 5.0], 4.4 [4.3 – 4.6], and 3.7 [3.5 – 4] arbitrary units (AU)/mL, respectively) to 4 weeks after vaccination (4.9 [4.8 – 5.3], 4.6 [ 4.4 – 4.8], and 3.9 [3.7 - 4], respectively; p >0.05 for all) (Fig1). H7 titers were ∼5-fold and ∼11-fold lower than vs H3 and H1, respectively. Median area under the curve (AUC) neutralization was similar between baseline and 4 weeks for H1N1 (1.5 [1.1 – 1.8] to 1.5 [1.1 – 2.4], p = 0.23) but significantly increased for H3N2 (0.03 [0 – 0.6] to 0.06 [0 – 0.5], p < 0.05) (Fig2). H3 binding was positively correlated with H1 (r = 0.75, p< 0.05) and H7 (r = 0.58, p< 0.05), but H1 and H7 titers did not correlate (r = 0.21, p >0.05) (Fig3). H1 binding and neutralization were positively correlated (r = 0.65, p< 0.05), but H3 binding and neutralization were not significantly correlated (r = 0.04, p >0.05).

**Conclusion:**

Anti-HPAI humoral immunity appears low in ISPs without substantial boosting by vaccination, raising concern for vulnerability to infection and disease. H3 binding may be associated with betters cross-reactivity with H7 than H1. Anti-H5 Ab evaluation is ongoing and of high importance given widespread H5N1 circulation and potential threat to ISPs.

**Disclosures:**

William Werbel, MD PhD, AstraZeneca: Advisor/Consultant|Novavax: Advisor/Consultant Andrew H. Karaba, MD PhD, GSK: Advisor/Consultant|Hologic: Advisor/Consultant

